# Modeling pesticides and ecotoxicological risk assessment in an intermittent river using SWAT

**DOI:** 10.1038/s41598-024-56991-6

**Published:** 2024-03-16

**Authors:** Marco Centanni, Giovanni Francesco Ricci, Anna Maria De Girolamo, Francesco Gentile

**Affiliations:** 1 Department of Soil, Plant and Food Sciences, University of Bari Aldo Moro, Bari, Italy; 2 Water Research Institute, National Research Council, Bari, Italy

**Keywords:** Environmental sciences, Hydrology

## Abstract

The present work aimed to predict the fate of two pesticides, copper (Cu) and glyphosate in a Mediterranean basin with an intermittent river and to assess the ecotoxicological risk related to their presence in water bodies coupling field measurements of streamflow and pesticide concentrations, and an eco-hydrological model. The Soil and Water Assessment Tool (SWAT) model was calibrated and, subsequently used to assess predicted environmental concentrations of pesticides in surface waters. The ecotoxicological risk related to the presence of Cu and glyphosate in surface water was assessed at the reach scale by using the Toxicity to Exposure Ratio approach (TER). Measurements of glyphosate concentrations (< 0.5 μg l^−1^) exceeded the maximum European threshold of environmental quality standards for pesticides (EQS) of 0.1 μg l^−1^. High concentrations of glyphosate were predicted in the wet season and in September, when glyphosate is mostly used in vineyards and olive grove productions. Acute risk (TER < 100) associated with the presence of glyphosate was detected for several reaches. High concentrations of Cu (< 6.5 μg l^−1^), mainly used as a fungicide in vineyards, were predicted in several river reaches. The results of the ecotoxicological risk assessment revealed that November and January were the critical months during which most of the river reaches showed a chronic risk associated with the presence of Cu.

## Introduction

Over the past century, the global population has swiftly increased, and in the current century, growth continues implying an increase in food needs^1,2^**.** In 2050, the population is projected to increase by 30% to approximately 9.2 billion^3,4^. Although, intensive agriculture was a tool to achieve a substantial increase in crop production, the high level of pesticide used is a threat to biodiversity, soil ecosystems, and water resources^5–7^ Pesticides are largely used in intensive agriculture because they contain active substances able to kill unwanted insects, harmful fungi, or weeds^8^. But, pesticides can also derive from urban wastewaters due to the treatment carried out in parks, gardens or along roads and railways^9^. An active substance is "persistent" if its half-life in water is more than two months and, in soil or sediments, if it is more than six months^10^. The transport of pesticides residue through drift, runoff and leaching can lead to contamination of waters and soils at the basin scale^11–15^ or global scale, through long-range atmospheric transport phenomena^16^. Indeed, once pesticides are applied to crops, a fraction is intercepted by the foliage and a fraction reach the soil surface ^17^. Then, through various processes including foliar wash-off by rain, surface runoff, soil erosion, or percolation into the aquifer, dissolved or sorbed pesticide can end up in water bodies ^5,9^. Other fraction could be lost through wind drift and volatilization^7,18^.

Several studies have shown that pesticides can affect the behaviour and physiology of aquatic organisms by hampering reproductive processes and reducing the entire community’s health^19–21^. Indeed, some compounds can cause reproductive and endocrine disruptions, inhibition of amino acid biosynthesis and neurological disturbances in fish and amphibians^9^**.** This effect could be enhanced in intermittent rivers, where the dilution effect is low due to the hydrological regime^22,23^.

The use of pesticides in agriculture is currently under debate. Indeed, notwithstanding the unintended effects, the abolition of pesticides could cause a reduction up to 40% of the crop yield^4^. The actual environmental strategies such as the *Transforming our world: the Agenda 2030 for Sustainable Development*^24^ and the *Farm to Fork* strategy of the European Green Deal^25^ aim to secure food production and make an appropriate use of pesticides. In this context, the monitoring of pesticides in soil and waters is fundamental especially for those compounds of particular interest (priority substances) due to the possible related ecotoxicological risk^26^. However, since direct measurement can be costly in terms of time and economic resources, several studies, that target the identification and toxicity of single or mixed pesticides, used available monitoring data^27,28^. Hence, together with field measurements, models may be fundamental tools for water resources managers to analyse the transport and fate of pollutants in water bodies and to find critical source areas^29^.

Several models such as MACRO^30^, PRZM (Pesticide Root Zone Model)^31^, SPIDER (Simulating pesticides in ditches to assess ecological risk)^32^ and SWAT (Soil and Water Assessment Tool)^33^ have been developed in recent decades to simulate the fate of pesticides^34,35^**.** SWAT is a spatially distributed and physically based model that was developed in the 1990s by the United States Department of Agriculture—Agricultural Research Service (USDA-ARS)^33^. This model is one of the most commonly used to simulate the fate and transport of pesticides at the basin level due to the possibility to evaluate the compound contamination considering different scenarios of Best Management Practices (BMPs)**,** climate and land use changes^36,37^. Many papers report SWAT model implementations to simulate hydrology^38^, sediments^39–41^, nutrients^42^, and pesticides^29,43^. However, few published papers reported studies on monitoring and modeling pesticide concentrations in small intermittent rivers^23^. Indeed, difficulties may arise when modeling hydrological regime and water quality of these rivers due to the limited data availability, and to the extremely low flow and zero-flow^44^**.**

The general aim of the present work was to develop a specific methodological approach to assess pesticide concentrations and the corresponding ecotoxicological risk in an intermittent river system in order to identify critical river reaches and timing. The specific objectives were (i) to quantify the predicted environmental concentration (PEC) and loads delivered to the semi-enclosed sea “Mar Piccolo” of Cu and glyphosate and, (ii) to identify the river reaches with Toxicity to Exposure Ratio (TER) beyond the threshold of ecotoxicological concern. The SWAT model ability in predicting pesticides in the surface waters was tested in a complex case study: the Canale d'Aiedda basin (SE Italy), which included karstic areas and limited data availability (e.g. streamflow, point source discharge, pesticide concentration in surface waters, pesticide application rate). The results will contribute to increase the knowledge concerning pesticide modeling (i.e. pesticides parameterization) in intermittent river systems. In addition, the methodological approach for assessing the ecotoxicological risk at the reach scale can give an important contribution to the decision-makers.

## Materials and methods

### Study area

**Figure 1:**
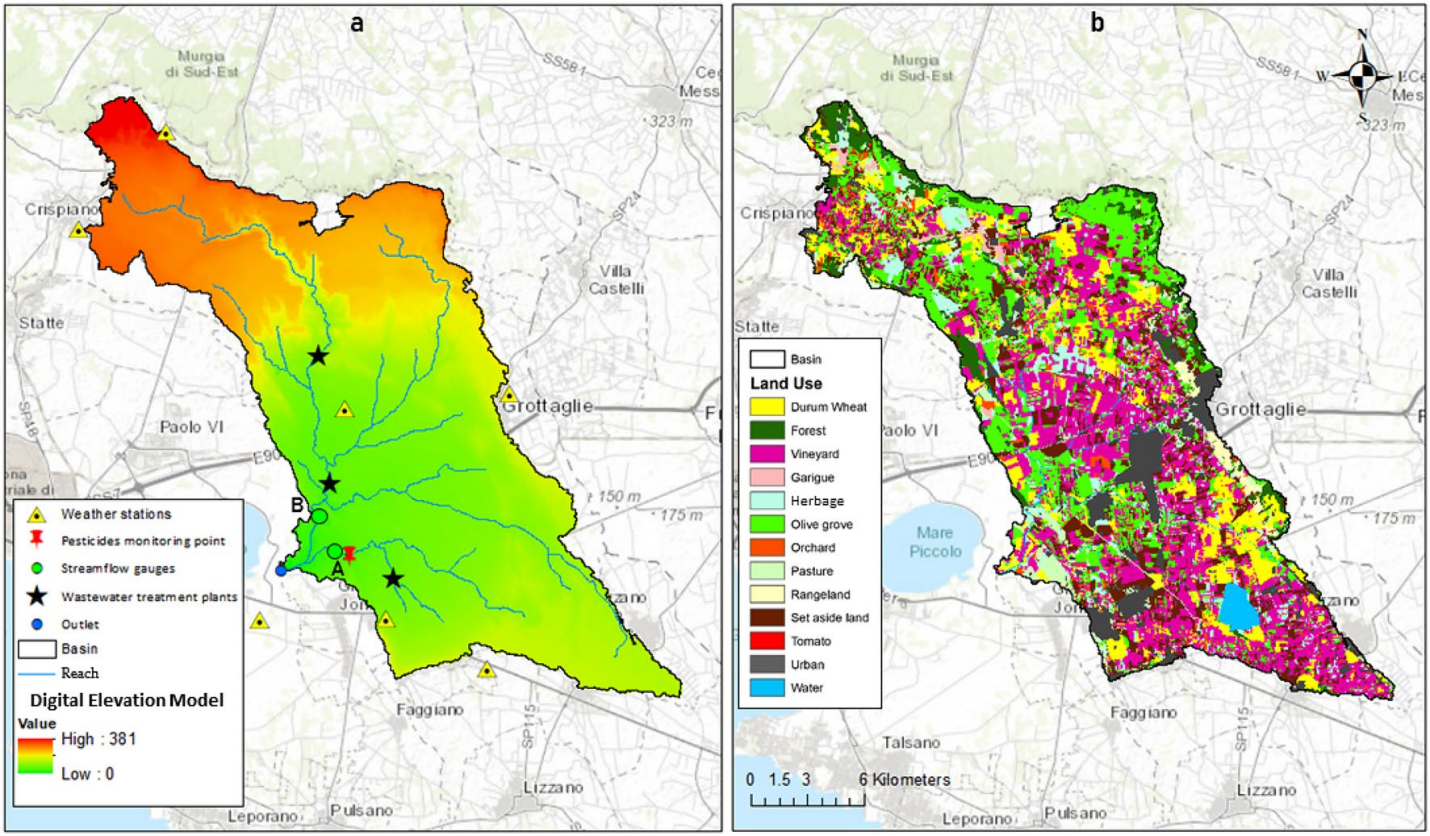
(**a**) Study area: Canale d’Aiedda basin, Apulia Region (SE, Italy). (**b**) Land use of Canale d’Aiedda basin. (QGIS version 3.4.13. https://www.qgis.org/it/site/).

The Canale d'Aiedda basin is located in the province of Taranto, in the Apulia region in southern Italy. The river flows into the semi-enclosed sea called “Mar Piccolo”. The banks and the bed of the river system are almost all covered by concrete. The hydrological regime is natural and intermittent in the upstream part of the basin, while it is almost perennial, in the remaining area, due to the presence of the discharges from three wastewater treatment plants (WWTPs), located in the municipalities of Montemesola, Monteiasi, and San Giorgio Ionico (Fig. 1a). The total drainage area, excluding the upstream karst formations, is 222 km^2^ (360 km^2^ with karst areas), the average altitude is 168 m a.s.l., between 0 and 517 m, and the average slope is 2.7 °^5^. The climate is Mediterranean and classified in the Koppen cold semi-arid climates class (BSk). The average annual rainfall (period 2000–2020; Grottaglie station) varies between 352 and 907 mm, and is characterized by intense and short events in summer and autumn. The average temperatures (period 2000–2020; Grottaglie station) are 5.2 ° in January and 32.8 ° in August. The soil textures vary from silty-clay to sandy loam. In the north-eastern edges of the basin, fractured limestone carbonate rocks, with a high water infiltration rate that recharges the deep groundwater aquifer, characterize the Canale d’Aiedda lithology. The particular hydrogeological structure generates a consistent groundwater discharge which flows in submarine springs (locally called “citri”)^46^. The basin is mainly agricultural (88.9%) while only 10% is urbanized. The vineyards cover an area of 36.3% the olive groves cover 24.5% and the durum wheat cover 28.1%. Among the other crops there are almond trees, grasses, tomato, orange groves, and arable land (i.e. pastures, set aside land). Natural areas, such as forests and rangeland cover 5% of the surface (Fig. 1b). Before to reach the Mar Piccolo, the river flows within the “Regional Natural Reserve Palude la Vela” which belongs to the Site of Community Importance (SIC) “Mar Piccolo” (IT9130004). Here the vegetation is characterized by Mediterranean maquis, mostly made up of myrtle, mastic, and holm oak which are able to offer shelter and food to many sedentary species (e.g. grey herons and finch) and migratory birds (e.g. flamingoes and curlews)^46–48^**.**

### Monitoring

**Figure 2:**
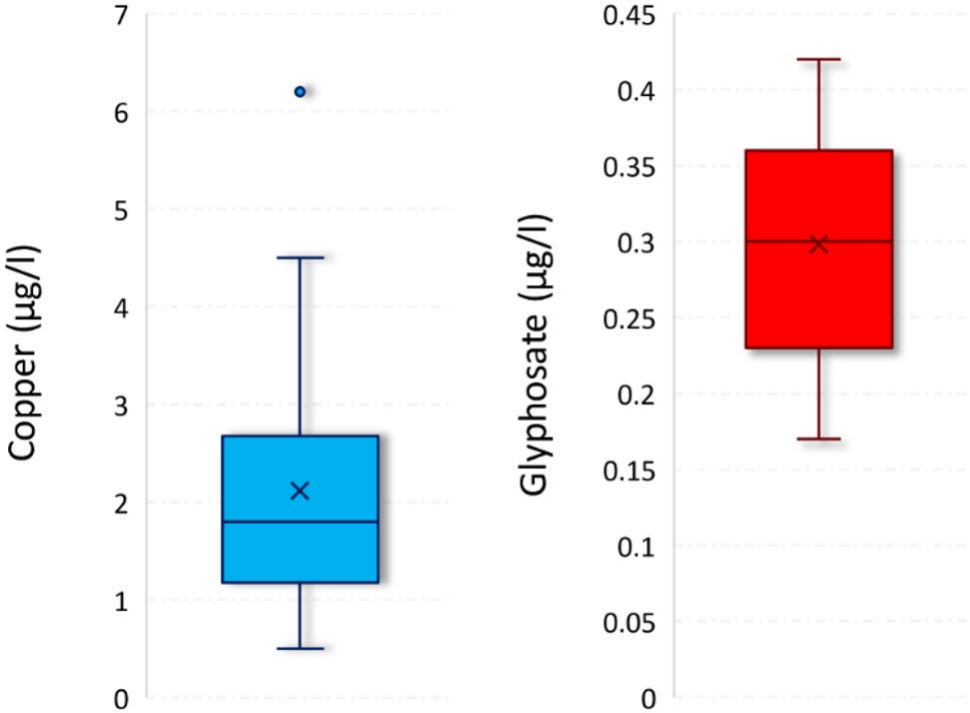
Observed copper and glyphosate concentrations (2021) at the Canale d’Aiedda (station A). In the plot: the median value is the horizontal central line, the mean value is the X; the 25th and 75th percentile values are the lines of box, the whiskers are the 5th and 95th, respectively; dot is an outlier. (Excel 365, https://www.office.com/).

Historical streamflow and water quality data were unavailable for the study area. In 2017, two flow measurement stations (MDS Dipper-PT, © 2019 Seba Hydrometrie, Kaufbeuren, Germany) were installed on the main course of the stream and programmed to monitor the streamflow on a sub-hourly time scale (Fig. 1a). Streamflow was monitored continuously from August 2017 to December 2019. Surface water samples for pesticide analyses were collected twice a month for a year (January to December 2021) very close to one of the hydrometric stations of the Canale d'Aiedda basin (Fig. 1a). For each sample a 1.5 L of water was collected in PET bottle and stored in a refrigerator. The analyses were carried out by an certified laboratory (methods 1495-CH-12 and 195-CH-39, for all compounds except for Cu) based on gas/liquid chromatography—mass spectrometry (limit of quantification < 0.5 μg l^−1^). The method used for Cu determination was the 11-A (UNI EN ISO 17294–2:2016; limit of quantification 0.5 μg l^−1^). A large number of compounds (i.e. 560 pesticides and 6 metabolites) were investigated. Only traces of Cu and glyphosate were found. Cu was quantified in the totality of samples with a median concentration value of 2.1 μg l^−1^ (maximum concentration 6.2 μg l^−1^). Glyphosate instead was quantified in only eight sample with a median concentration of 0.29 μg l^−1^ (maximum concentration value of 0.42 μg l^−1^) (Fig. 2).

### SWAT model

In this work, the hydrological and water quality model SWAT was used^33^. The model divides the basin area into sub-basins and in turn into Hydrological Response Units (HRU), the basic unit for the water balance calculations, which are based on defined thresholds referring to land use, topography, and soil properties^17^. The surface runoff is computed with the Soil Conservation Service Curve Number method (SCS-CN)^49^, while landscape and the in-stream sediment are obtained through the Modified Universal Soil Loss Equation MUSLE^50^ and the Bagnold stream power Equation^51^, respectively. The Hargreaves^52^, Penman–Monteith^53^ and Priestly–Taylor^54^ methods can be used to estimate the potential evapotranspiration^17^. The movement of the pesticide in the land phase (e.g. wash-off, degradation and leaching) is computed by equations derived from GLEAMS^55^. Specifically, the model considers that a fraction of the pesticide can be intercepted by the leaves, and a fraction can reach the soil, both through direct application or leaves wash-off. The latter process will occur when the rainfall of a given day exceeds 2.54 mm^17^. Moreover, a part of pesticide can be degraded in both soils and leaves. The amount of degraded pesticides depends on the number of days required to reduce the concentration of a given pesticide by half, or the half-life. Pesticides characterized by high solubility in water and low soil adsorption coefficient normalized for soil organic carbon (SKOC) can percolate into soil profiles and, consequently into the groundwater system^17^. Soluble and sorbed pesticides which move from the land to the stream, through runoff lateral flow and percolation, are calculated by algorithms taken from EPIC^50^. Then, the processes which involve the transformation and the transport of pesticides in streams, such as degradation, volatilization, settling and resuspension, are simulated by using mass balance equations implemented by Chapra^56^. SWAT allows to simulate only one pesticide at a time for runs which have to be activated through the IRTPEST function in the .bsn file^17^. Model results can be assessed at different spatial (e.g. basin, subbasin and reach scale) and temporal (e.g. daily, monthly, yearly) scales^17,44^.

### SWAT model setup

Due to the position of the karst areas and to the absence of flow within the river network, confirmed by field surveys carried out periodically in the studied period, the karst areas were cut out of the basin boundary, since they were considered as not contributing to the streamflow^57^.

The river basin was divided into 40 sub-basins and 271 Hydrologic Response Units (HRUs) discretized through thresholds of 10%, 10%, and 20% of land use, soil classes, and slopes, respectively. SWAT was run from 1997 to 2021, on a daily time scale, including a three-year warm-up period. The Hargreaves method was used to calculate PET, while the SCS-CN method was used for surface runoff^45^.

As reported in table A1 (supplementary file), the following inputs were included to set up the model: topography (*DEM*) land use, soil map, soil properties, agricultural management practices and pesticide applications, weather data. Concerning WWTPs, volumes and water quality data on the monthly time scale were collected from Regional Agency for Environmental Protection and Apulian Water Authority.

Data from 7 meteorological stations (Fig. 1a) from 1997 to 2021 on a daily time scale were used. For the same period of time, inlets from three wastewater treatment plants (WWTPs) were included. Agricultural practices (i.e. fertilization, tillage, pesticide application, and irrigation) were included into the management database of SWAT using data retrieved from direct interviews and from the agricultural census^46^. Twenty-one classes for land use and eleven for soil type were identified in the basin. Three shallow tillages were applied in olive groves. Fertilizers were spread in April (urea and 12-8-8) and in August (13-46-00). A total amount of 500 m^3^ha^−1^ of water was given to the crop from June to September. Two shallow (10 cm) operations were applied for the vineyards in February and May, while one deep (35 cm) tillage was applied in October. Fertilizer were spread in February (12-12-17 and 10-5-15), October and in November (manure for both). A total amount of 2400 m^3^ha^−1^ of water was given to the crop from May to September. A three-year rotation was considered for durum wheat (durum wheat–herbage–set aside). For the durum wheat, a deep and a shallow tillage operation were applied in August and October, respectively. Fertilizers were spread in December (25-15-00) and in February (urea). For orchards and for the other minor land uses, for which data were unavailable, a deep and a shallow tillage operation were applied in spring and autumn, respectively, and the irrigation and the fertilization were set as automatic^45^. Due to the absence of conservative practices in the study area the USLE P (Universal Soil Loss Equation—Support practice factor) factor was set to 1^45,47^.

For pesticides it was assumed that Cu sulphate (98%) was the main copper-based pesticide applied in vineyards as fungicide, olive groves as bactericide, and orchards (i.e. citrus fruits) as fungicide. Since no reliable data on Cu application (amount) were available, as farmers didn't provide precise information, the application rates were assumed based on the current European regulations, which authorize a total maximum amount of 28 kg of Cu per hectare over 7 years^58^. Specifically, for olive groves, treatments were carried out in spring (April) and after the harvest in Autumn–Winter (September, October, November, December and January depending on the harvest period). Also in the vineyards, the treatments were carried out in Spring (April, May and June) and after the harvest (October and November depending on the harvest period). In the other orchards, treatments were carried out in Autumn–Winter (October, November, December and January) and in spring (May). Monthly loads of Cu in the effluent from the WWTPs were also included in the model setup (*source: *http://www.arpa.puglia.it/web/guest/depuratori).

Roundup (granular soluble in water), whose active substance is glyphosate, is a systemic herbicide to be used for the control of monocotyledonous and dicotyledonous weed species (annual, biennial, or perennial). For vineyard productions it was applied in spring (March, April) in pre-flowering, at the end of spring in suckering (May), and in mid-November, December to avoid re-infestation. For olive groves, it was applied near flowering (end of March), or for pre-harvest (September) weeding of pitches. In addition, glyphosate was applied also in durum wheat (April, July and October) and orchard cultivations (February, March and October). Specifically, on durum wheat, glyphosate was applied in pre-seeding because if applied before maturity, it can accumulate in the development of the grains sufficiently to affect germination^59^. Due to the absence of data on the applied amount, an application rate of 2.28 kg ha^-1^ of the active substance (equivalent to 3.16 kg product ha^-1^) was considered following the Roundup safety data sheet.

Data regarding the glyphosate attribute (i.e. Koc, wash off fraction, foliar and soil half-life and water solubility) were included in the SWAT database. For the Cu the model was adapted by adding the compound in the pesticide database. The main attributes were retrieved from the research carried out by Serpa et al.^36^ and from the International Union of Pure and Applied Chemistry (IUPAC) website (http://sitem.herts.ac.uk/aeru/iupac/Reports/178.htm).

### Model calibration

The model was calibrated for hydrology, using continuous measurements from both the gauging stations (A and B), from August 2017 to December 2019 (at the daily time scale), and for pesticides by using discrete sampling carried out during 2021 close to gauge A (Fig. 1). The SWAT-CUP (SWAT-Calibration and Uncertainty Programs) tool by means of the Sequential Uncertainty Fitting (SUFI-2) algorithm was used for the sensitivity analysis and for both the calibration processes^60^. For both hydrology and pesticides, only the calibration was performed. This strategy was adopted in order to include the different hydrological conditions (wet and dry) and, because of the limited number of measured data^45^. Additional details about the hydrological calibration are reported into Ricci et al.^45,57^. The set of parameters (Table 2) used for the pesticide calibration was retrieved from both the literature and from the SWAT manual^43,61,62^. Initial values of some parameters such as “Degradation half-life of the chemical on the soil” (HLIFE_S), “Degradation half-life of the chemical on the foliage” (HLIFE_F), “Wash off fraction” (WOF), “Application efficiency” (AP_EF), “Solubility of the chemical in water” (WSOL), and SKOC were derived for Cu from the research carried out by Serpa et al.^36^ and from the IUPAC website (http://sitem.herts.ac.uk/aeru/iupac/Reports/178.htm), and for glyphosate from the pesticide appendix of the SWAT manual^61,63^. Model performance was assessed by using Nash–Sutcliffe efficiency (NSE), percent bias (PBIAS), and coefficient of determination (R^2^). In general, the model simulation was assumed as “satisfactory” if NSE > 0.50 and R^2^ > 0.50, and if PBIAS ± 25% and as “good” if NSE > 0.65, R^2^ > 0.60 and PBIAS ± 15 for streamflow; meanwhile for pesticides, the same value suggested for nutrients or PBIAS ± 70% (satisfactory) and PBIAS ± 40% (good) were considered^64^.

### Ecotoxicological risk assessment

The ecotoxicological risk related to the presence of Cu and glyphosate in surface water was assessed at the reach scale by using the TER approach^65^ for the chronic and the acute risks. The chronic and the acute risks can be defined as the “adverse effects on any living organism in which symptoms develop slowly over a period of time (often the lifetime of the organism) or reoccur frequently” and the “adverse effects on any living organism that results from a single dose or single exposure of a chemical”, respectively^66^.

A two-step approach was adopted, first the TER for chronic risk was evaluated, then the TER for acute risk.

The TER for chronic risk was evaluated with the following equation (Eq. 1)^65^:

1 $$TER=\frac{NOEC}{PEC} \le 10$$

where: NOEC is the *No Observed Effect Concentration*, which is defined as “the highest concentration tested at which the substance is observed to have no statistically significant effect (p < 0.05) when compared with the control, within a stated exposure period”^67,68^;

PEC is the Predicted Environmental Concentration, which is provided by the model at the reach scale^67,68^.

The TER for acute risk was evaluated by using the following equation (Eq. 2)^65^:

2 $$TER=\frac{{L(E)C}_{50}}{PEC} \le 100$$

where: L(E)C_50_ is LC_50_ (“the toxicant or effluent concentration that would cause death in 50% of the test organisms”) or EC_50_ (“median effective concentration at which 50% of the test organisms die”). Both LC_50_ or EC_50_ can be used depending on the available data^67,68^.

Following the European Commission Regulation 546/2011^65^, the chronic risk was assessed for algae, daphnia and fish, while the acute risk for daphnia and fish. Moreover, the most sensitive aquatic species (i.e. lower values of NOEC or L(E)C_50_) were also considered if different from the species indicated by the Regulation^65^. NOEC and L(E)C_50_ were derived from the IUPAC database (http://sitem.herts.ac.uk/aeru/iupac/Reports/178.htm, http://sitem.herts.ac.uk/aeru/iupac/Reports/373.htm). Specifically, to assess the chronic risk for glyphosate, the NOEC was 1000 μg l^−1^ (Fish—*Brachydanio rerio*—Chronic 21 day), 12,500 μg l^−1^ (Daphnia—*Daphnia magna*—Chronic 21 day) and 2000 μg l^−1^ (Algae—*spp*—Chronic 96 h NOEC). To assess the acute risk, the L(E)C_50_ was 100,000 μg l^−1^ (Fish—*Oncorhynchus mykiss*—Acute 96 h), 100,000 μg l^−1^ (Daphnia—*Daphnia magna*—Acute 96 h) and 10,000 μg l^−1^ (Sediment dwelling organisms – *Chironomus plumosus*—Acute 96 h). For the chronic risk for Cu the NOEC was 970 μg l^-1^ (Fish—*Oncorhynchus mykiss*—Chronic 21 day) and 57 μg l^-1^ (Daphnia—*Daphnia magna*—Chronic 21 day). No value for algae was reported. For the acute risk the L(E)C_50_ was 13,200 μg l^−1^ (Fish—*Oncorhynchus mykiss*—Acute 96 h) and 2300 μg l^−1^ (Daphnia—*Daphnia magna*—Acute 96 h). The risk was considered unacceptable when TER < 10 for chronic exposure and TER < 100 for acute exposure^65^. Maps were designed for the chronic risk, while graphs were reported for the acute risk accordingly with the results.

## Results

### Hydrological calibration

**Figure 3:**
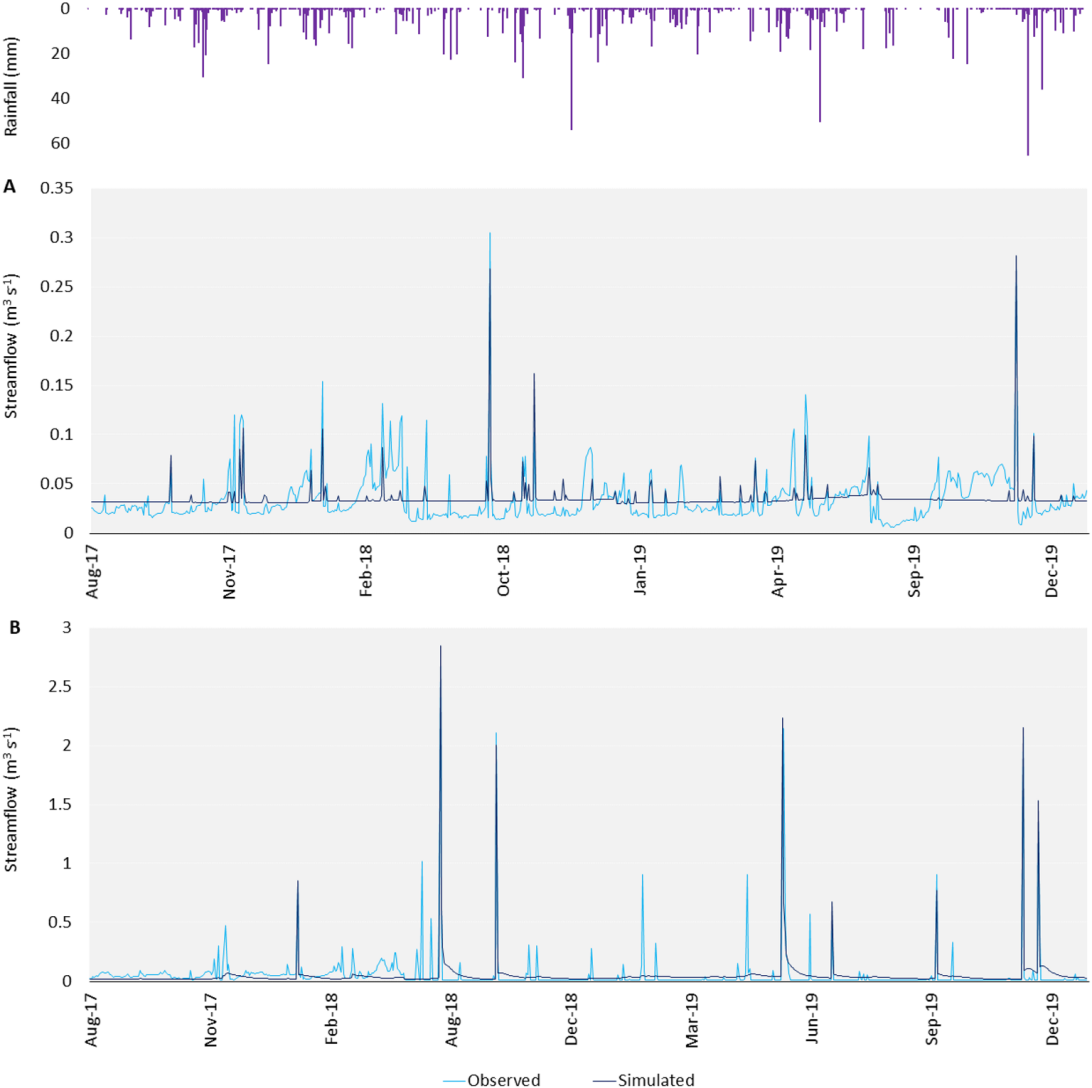
Observed and simulated streamflow for the calibration period: (**A**) gauge A; (**B**) gauge B.

The results of calibration, considering the statistical performance, were satisfactory for gauge (A) with R^2^ R^2^ = 0.48, NSE = 0.47, and PBIAS = − 4.2% and good for gauge (B) with R^2^ = 0.72, NSE = 0.71, and PBIAS =  + 5.1% (Table 1). The streamflow was overestimated in gauge A (PBIAS = − 4.21) and underestimated in gauge B (PBIAS =  + 5.05) (Table 1)^45,57^. For both the gauging stations the SWAT model simulated well the main peaks (i.e. gauge A: the highest measured peak was 0.27 m^3^s^−1^ and the simulated peak was 0.31 m^3^s^−1^; gauge B: the highest measured peak was 2.67 m^3^s^−1^ and the simulated peak was 2.85 m^3^s^–1^), the normal flow (0.095 m^3^ s^−1^ > Q > 0.012 m^3^ s^−1^) was underestimated (i.e. gauge A: Q_obs_ = 0.04 m^3^s^–1^ and Q_sim_ = 0.03 m^3^s^–1^; at gauge B: Q_obs_ = 0.05 m^3^s^−1^ and Q_sim_ = 0.04 m^3^s^–1^), and the low flow (Q < 0.012 m^3^ s^−1^) was generally overestimated (i.e. gauge A: Q_obs_ = 0.015 m^3^s^−1^ and Q_sim_ = 0.03 m^3^s^−1^; gauge B: Q_obs_ 0.008 m^3^s^−1^ and Q_sim_ = 0.02 m^3^s^−1^) (Fig. 3A**; **Fig. 3B)^45,57^. At the basin scale the modelled surface runoff (118.52 mm) was 19.3% of the modelled precipitation (612.80 mm), while the modelled total water yield (146.84 mm; Surface runoff + baseflow + lateral flow) was 25%, and modelled transmission losses (41.99 mm) were 6.8%. The modelled potential evapotranspiration was 1186.4 mm.

**Table 1:** Calibrated parameters for the best-fit simulation of streamflow at the gauge A and gauge B.

Parameter	Description	Initial value A;B	Calibrated value A	Calibrated value B
v__EVRCH.bsn	Reach evaporation adjustment factor	1;1	0.87	0.87
v__SURLAG.bsn	Surface runoff lag time	4;4	11.95	11.95
v__TRNSRCH.bsn	Portion of transmission losses from main channel that enter deep aquifer	0;0	0.53	0.53
r__CN2.mgt	SCS runoff curve number	**79–38**	-0.26 **60—29**	0.21 **98—48**
v__BIOMIX.mgt	Biological mixing efficiency	0.2;0.2	0.70	0.62
v__ALPHA_BF.gw	Baseflow alpha factor (days)	0.01;0.01	0.004	0.29
v__GW_DELAY.gw	Groundwater delay (days)	31;31	43.82	42.05
v__GWQMIN.gw	Threshold depth of water in the shallow aquifer needed for return flow to occur (mm)	1000;1000	1386.11	2222.12
v__REVAPMIN.gw	Threshold depth of water in the shallow aquifer for “revap” to occur (mm)	750;750	410.45	749.15
v__RCHRG_DP.gw	Deep aquifer percolation fraction	0.05;0.05	0.10	0.85
v__GW_REVAP.gw	Groundwater “revap” coefficient	0.02;0.02	0.19	0.18
r__SOL_K(..).sol	Saturated hydraulic conductivity	**26.90–0.04**	0.08 **30.11–0.06**	− 0.11 **24.82–0.05**
r__SOL_AWC(..).sol	Available water capacity of the soil layer	**0.117–0.08**	0.22 **0.15–0.09**	− 0.10 **0.105–0.07**
v__SOL_ALB(..).sol	Moist soil albedo	0.2;0.2	0.17	0.22
r__SOL_Z(..).sol	Depth from the soil surface to the bottom of the layer	**1969.41;29.52**	0.10 **2188.24–32.81**	− 0.23 **1531.77–22.97**
v__EPCO.hru	Plant uptake compensation factor	1;1	0.51	0.89
v__ESCO.hru	Soil evaporation compensation factor	0.95;0.95	0.23	0.68
v__CANMX.hru	Maximum canopy storage – vineyard	0;0	3.41	4.14
v__CANMX.hru	Maximum canopy storage – olive groves	0;0	4.26	5.37
v__CANMX.hru	Maximum canopy storage – durum wheat	0;0	1.77	0.32
v__CANMX.hru	Maximum canopy storage – garigue	0;0	2.37	2.22
v__CANMX.hru	Maximum canopy storage – rangeland	-;0	–	0.07
v__CANMX.hru	Maximum canopy storage – pasture	-;0	–	2.33
v__CANMX.hru	Maximum canopy storage – mixed forest	-;0	–	3.29
v__CANMX.hru	Maximum canopy storage – deciduous forests	-;0	–	4.30
v__CH_K1.sub	Effective hydraulic conductivity in tributary channel alluvium	0;0	107.08	29.67
v_CH_N1.sub	Manning’s “n” value for the tributary channels	0.014;0.014	17.90	10.07
v__CH_K2.rte	Effective hydraulic conductivity in main channel alluvium	0;0	7.02	43.23
v__CH_N2.rte	Manning’s “n” value for the main channel	0.014;0.014	0.25	0.28

v_ corresponds to the replacement of the original value with the new values reported in the row; r_ corresponds to the multiplication of the original values by 1 + the value reported in the row.

Values in bold represent the final range of the parameter^57^.

v_ corresponds to the replacement of the original value with the new values reported in the row; r_ corresponds to the multiplication of the original values by 1 + the value reported in the row. Values in bold represent the final range of the parameter^57^.

For the hydrological calibration some parameters, such as the Curve Number (CN2.mgt), the factor which controls the transmission losses from main channel (TRNSRCH.bsn), the hydraulic conductivity of the main channel (CH_K2), the deep aquifer percolation (RCHRG_DP) and the Manning’s roughness (CH_N2) of the main channel, were very sensitive and, therefore fundamental for the process^45^.

### Chemicals calibration

**Figure 4:**
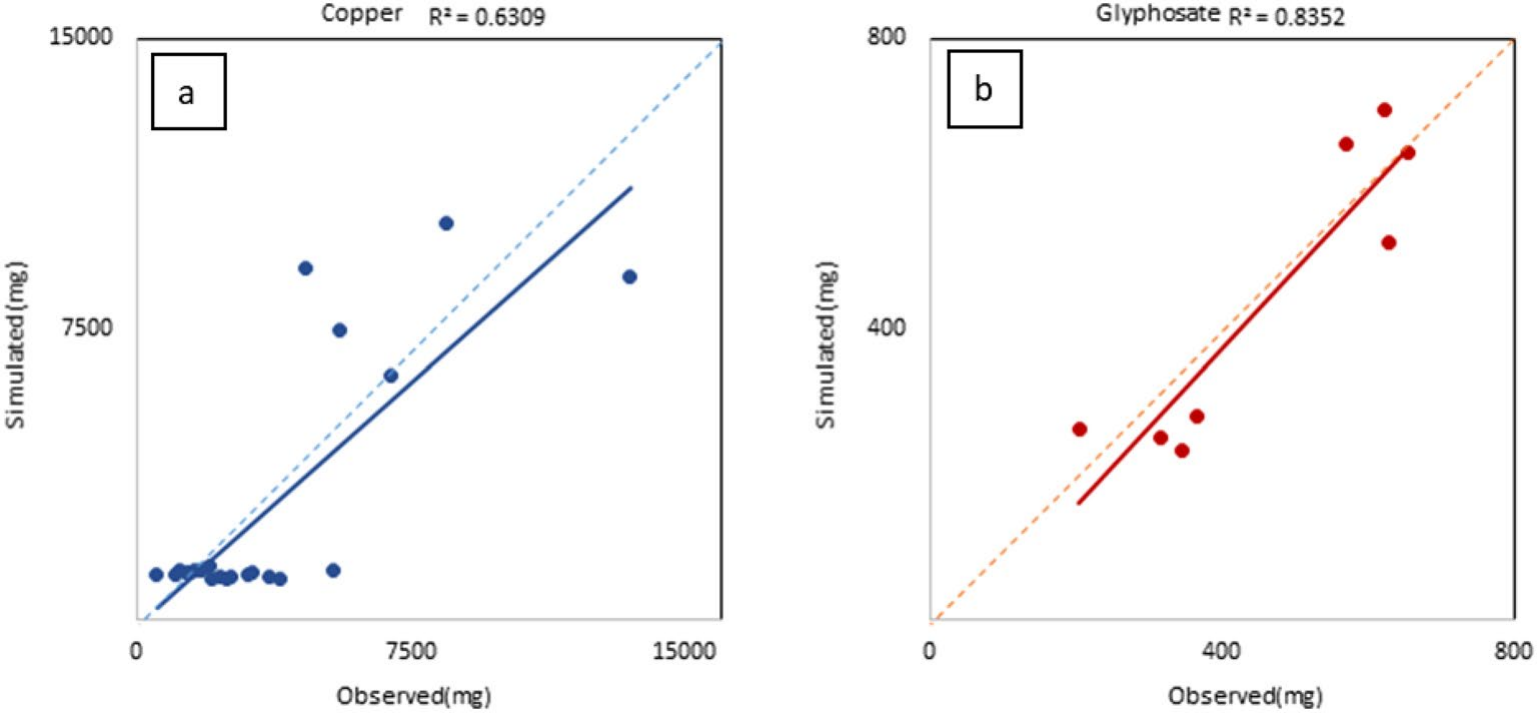
Comparison between daily simulated and observed loads: (**a**) Copper; (**b**) glyphosate.

The lists of the parameters used for glyphosate and Cu calibration and their fitted values are reported in Table 2. The results of the calibration in terms of statistical analysis were satisfactory for Cu with R^2^ = 0.63, NSE = 0.50, and PBIAS = 21.6%, and good for glyphosate with R^2^ = 0.78, NSE = 0.57, and PBIAS = 6.8%. However, the loads, estimated by the SWAT model were underestimated for both Cu (PBIAS = 21.6%) and glyphosate (PBIAS = 8.7%) (Fig. 4).

**Table 2:** Calibrated parameters, description, and their fitted values.

Parameter	Description	Initial value Copper;Glyphosate	Calibrated value Copper	Calibrated value Glyphosate
v__HLIFE_F.pest.dat	Degradation half-life of the chemical on the foliage (days)	2500;2.5	2500	2.5
v__HLIFE_S.pest.dat	Degradation half-life of the chemical on the soil (days)	10,000;47	10,000	161
v__SKOC.pest.dat	Soil adsorption coefficient normalized for soil organic carbon (mg/kg)/(mg/l)	115;884	94.05	17.21
v__WOF.pest.dat	Wash off fraction	0.5;0.6	0.029	0.97
v__AP_EF.pest.dat	Application efficiency	0.75;0.75	0.79	0.98
v__WSOL.pest.dat	Solubility of the chemical in water (mg/l)	3.42;900,000	3.42	100,000
v__PERCOP.bsn	Pesticide percolation coefficient	0.5;0.5	0.81	0.98
v__CHPST_REA.swq	Pesticide reaction coefficient in reach (day^−1^)	0.007;0.007	0.0003	0.0002
v__CHPST_VOL.swq	Pesticide volatilization coefficient in reach (m/day)	0.01;-	0.006	-
v__CHPST_KOC.swq	Pesticide partition coefficient between water and sediment in reach (m^3^/g)	0;0	0.0015	0.004
v__CHPST_STL.swq	Settling velocity for pesticide sorbed to sediment (m/day)	1;1	8.92	2.37
v__CHPST_RSP.swq	Resuspension velocity for pesticide sorbed to sediment (m/day)	0.002;0.002	0.83	0.009
v__CHPST_MIX.swq	Mixing velocity (diffusion/dispersion) for pesticide in reach (m/day)	0.001;0.001	0.019	0.0085
v__SEDPST_CONC.swq	Initial pesticide concentration in reach bed sediment (mg/m^3^ sediment)	0;0	963.91	106
v__SEDPST_REA.swq	Pesticide reaction coefficient in reach bed sediment (day^−1^)	0.05;0.05	0.0017	0.098
v__SEDPST_BRY.swq	Pesticide burial velocity in reach bed sediment (m/day)	0.002;0.002	0.0019	0.0019
v__SEDPST_ACT.swq	Depth of active sediment layer for pesticide (m)	0.03;0.03	0.049	0.049
v__SPCON.bsn	Linear parameter for calculating the maximum amount of sediment that can be reentrained during channel sediment routing	0.0001;0.0001	0.0033	0.014
v__SPEXP.bsn	Exponent parameter for calculating sediment reentrained in channel sediment routing	1;1	1.20	1.33
v__ADJ_PKR.bsn	Peak rate adjustment factor for sediment routing in the subbasin (tributary channels)	1;1	0.5	0.65
v__LAT_SED.hru	Sediment concentration in lateral and groundwater flow (mg/l)	0;0	1453.4	4027.5

v_ corresponds to the replacement of the original value with the new values reported in the row.

v_ corresponds to the replacement of the original value with the new values reported in the row.

The most sensitive parameters for both Cu and glyphosate were the soil adsorption coefficient normalized for soil organic carbon (SKOC.dat), the application efficiency (AP_EF.dat), the pesticide percolation coefficient (PERCOP.bsn) and the reaction coefficient in reach bed sediment (SEDPST_REA.swq). In addition, parameters related to the sediment routing (i.e. SPCON.bsn, SPEXP.bsn, ADJ_PKR.bsn, and LAT_SED.hru) resulted sensitive for Cu and the half-life of the chemical into the soil (HLIFE_S) was sensitive for glyphosate.

### Predicted environmental concentration (PEC)

**Figure 5:**
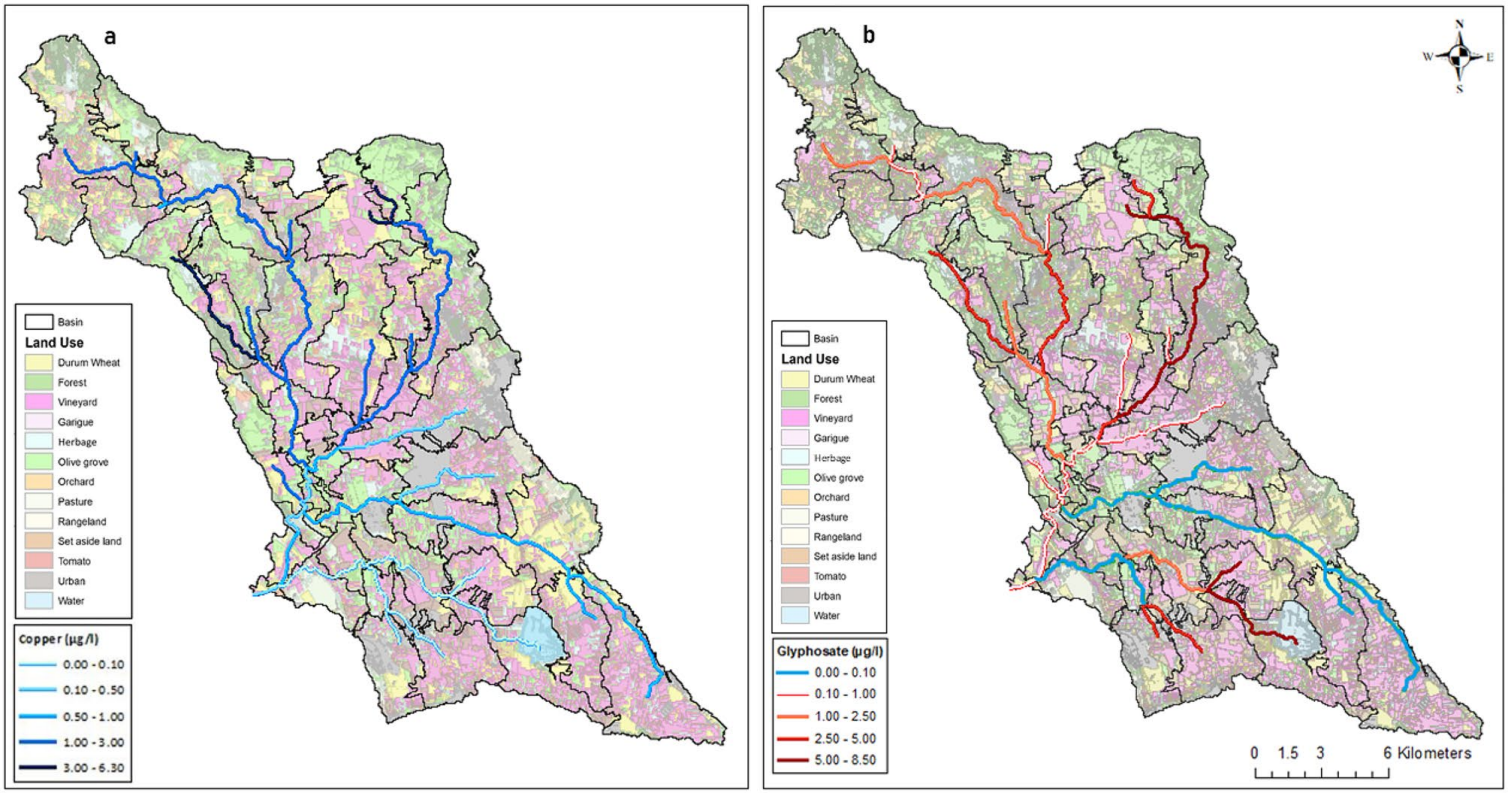
Predicted Environmental Concentration (PEC) for Cu (**a**) and glyphosate (**b**) for the year 2021. The value 0.1 μg l^−1^ was the Environmental Quality Standard (EQS) for glyphosate. (QGIS version 3.4.13. https://www.qgis.org/it/site/).

In 2021, the results of the SWAT model showed that the mean annual PEC of Cu ranged from 0.00 μg l^−1^ to 245 μg l^-1^ in the reaches defined in the river network (Fig. 5a) (highest value in reach 9, Fig. A1 supplementary file). The maximum PEC of Cu was modeled in the reaches located in the northern part of the area, corresponding to sub-basins where vineyards, olive groves, and durum wheat were the main agricultural productions (Fig. 5a) and main soil textures were clay and sandy-clay (Fig. A2 supplementary file). At the basin outlet, the annual load in surface waters, delivered to the Mar Piccolo predicted by the model was 69.45 kg y^−1^, corresponding to 0.08% of the total amount applied on the fields. These results suggest that net of drift losses, the most of the Cu applied is in soil and it can be both lost by leaching or involved in settling and resuspension processes. For Cu no Environmental Quality Standard (EQS) were fixed neither by the national regulations nor by the European Directives. The EQS defines a threshold value below which no adverse impact on the human health or on the environment occur. At the European level, Directive 98/83/EC has set a value of 2000 μg l^−1^, as the threshold limit for drinking water, meanwhile, the Italian Legislative Decree 31/2001, and subsequent amendments defined a more restrictive value equal to 1000 μg l^−1^. The PEC of Cu in the totality of the river reaches was always below this threshold.

In the same period, the mean annual PEC of glyphosate predicted by the model in the reaches ranged from 0.00 μg l^−1^ to 8.50 μg l^−1^ (Fig. 5b) (highest value in reach 33, Fig. A1 supplementary file). The annual load delivered to the Mar Piccolo was 71.94 kg y^−1^ corresponding to 0.1% of the applied amount. Net of wind drift losses, glyphosate mostly accumulates in the top-soil layers, and it may be lost through suspended sediment^69^ .

In several reaches, the PEC of glyphosate exceeded the EQS fixed for generic pesticides (of 0.1 μg l^-1^) by the European Union (EU) Directive (EU) 2020/2184^70^ and by the Italian Decree 172/2015^71^. It is a legally binding limit for individual substances, mainly used in Europe. Around the world, there is no unique definition. For instance, in the USA and Canada, the terms Ambient Water Quality Criteria and Water Quality Guidelines are used, respectively^72^. The SWAT model simulated the highest PECs of glyphosate in the river reaches located in the upstream subbasins of the study area, where the main crops were vineyards and olive groves, durum wheat, and ryegrass (Fig. 5b) and main soil textures were clay, clay-loam and sandy-clay (Fig. A2 supplementary file).

### Ecotoxicological risk assessment

**Figure 6:**
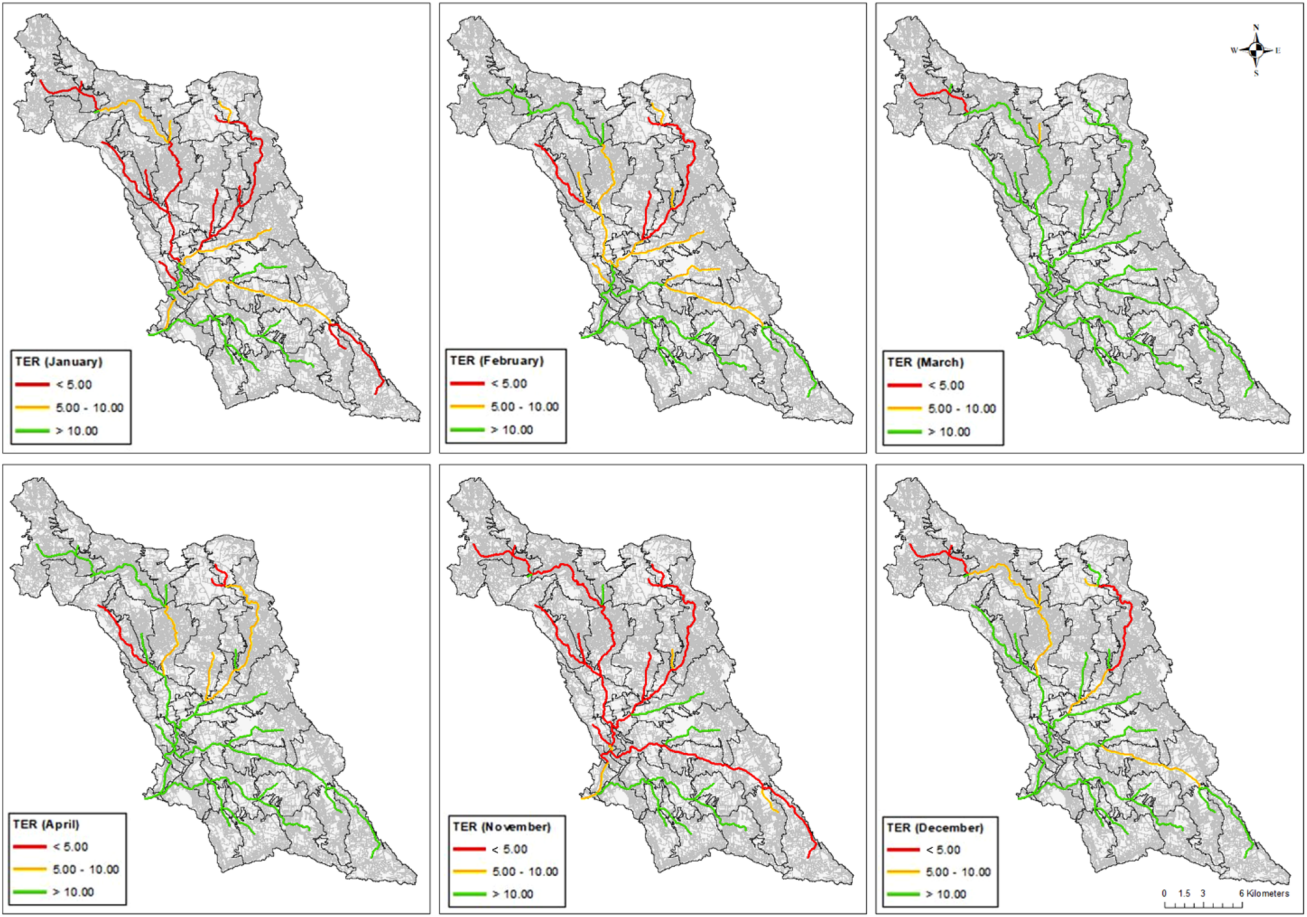
Maps of the monthly chronic toxicity to Exposure Ratio (TER) for the copper for fish (Oncorhynchus mykiss) (2021). Red and yellow indicate the reaches under high exposure. (QGIS version 3.4.13. https://www.qgis.org/it/site/).

The chronic ecotoxicological risk was evaluated, for both compounds, on a monthly scale for the year 2021. Figure 6 reports the maps of the months in which the ecotoxicological risk for the Cu for fish was detected for at least one reach of the river network. November and January were the critical months during which much of the river reaches showed TER < 10, which means chronic exposure. For daphnia instead, since the NOEC was much lower than the one for fish, most of the months showed a TER < 10 for at least one river reach. January, November and December were the most critical months (Fig. A3). This result was mainly due to the combination of the two factors: the high surface runoff that occurred during those months and the Cu applications. For fish during the dry period (from May to October), the river did not show an ecotoxicological risk associated with the presence of Cu in the water column. Indeed, from May to October, the rainfall amount and surface runoff are very low within the basin, therefore also the amount of Cu delivered to the stream is low. A chronic ecotoxicological risk was instead identified for daphnia (Fig. A3).

The results for the acute risk associated with the presence of Cu showed TER values < 100 for fish in almost all the river reaches in January, November, and December. During these months Cu was largely applied on olive groves, vineyards and orchards and high rainfall occurred. The river was not at risk in May, June, and July (Tab A2). The river reaches at risk were located in the same areas where high PEC values were found (Fig. 5a). The duration of the overruns was mostly lower than 24 h (h). Details about the reaches and frequencies of occurrence of acute risk are reported in table A2 (supplementary material S1). Figures A4a–c report an example of three events in which TER was found < 100 of durations 72 h, 48 h, and 24 h, respectively. The figure refers to three different reaches (22, 34, and 32; Fig. A1 supplementary file). For daphnia TER was < 100 for the whole period, with a duration of the overrun mostly higher than 96 h.

**Figure 7:**
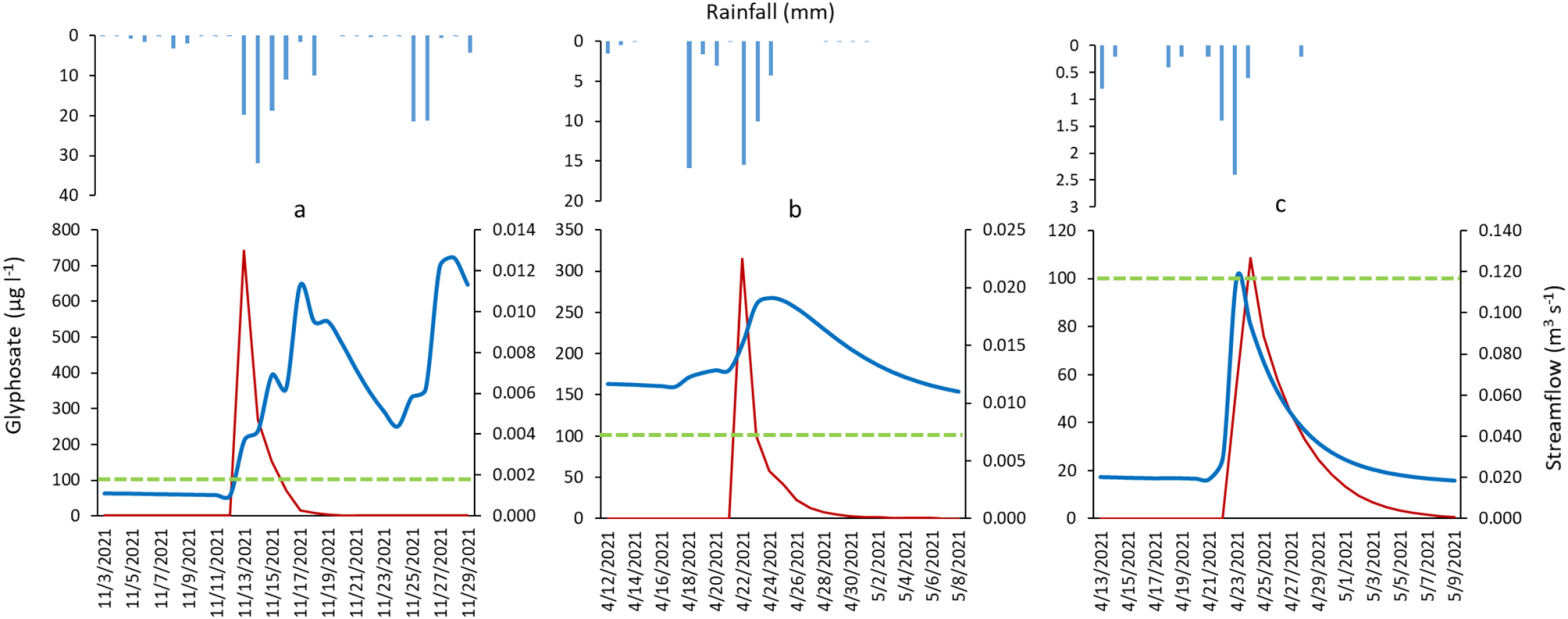
Concentration for glyphosate (red line), streamflow (blue line) and precipitation for three events in 2021. The green line represents the concentration limit for the acute risk for sediment dwelling organisms (*Chironomus plumosus*). (**a**) reach 6, 72 h, (**b**) reach 13, 48 h and (**c**) reach 8 24 h. A map of the reaches is reported in Fig. A1 in the supplementary file.

For glyphosate no chronic risk was detected based on the model results, neither for fish daphnia and algae. The results for the acute risk showed no risk for fish and daphnia and TER values < 100 for sediment dwelling organisms in March, April, November and January. During these months glyphosate was applied in most land uses (Olives, wheat and vineyard) and high rainfall occurred. The river reaches in which acute risks was detected were located in the same areas in which the PEC exceeded the EQS (Fig. 5b). The duration of these overruns was generally lower than 24 h. Details about the reaches and frequencies of occurrence of acute risk are reported in table A3 (supplementary material S1). Figure 7a–c report an example of three events in which TER was found < 100 of durations 72 h, 48 h and 24 h, respectively. The figure refers to three different reaches (6, 13 and 8; Fig. A1 supplementary file). The most important factor influencing the glyphosate peaks of concentration is the streamflow. As Fig. 7a,b show the peak of concentration precedes the peak of streamflow, contrarily in Fig. 7c the peak of streamflow precedes the peak of glyphosate. This is due to the fact that in the first case the area contributing to the drainage is small and close to the subbasin outlet, while in the second case the contribution of pesticides comes from remote areas (Fig. A1 supplementary file). In a sequence of flood events, the peak of glyphosate was not simulated in the second flood (Fig. 7a) suggesting a rapid dissipation of the substance.

## Discussion

The application of the eco-hydrological models in Mediterranean basins with intermittent streams can be particularly difficult^39,44,45^. Working with limited data or with low concentrations of pollutants, which are common characteristics of small river basins in the Mediterranean Region, complicates the implementation and calibration of models^73^. For this reason, it is very important to accurately build a methodological approach as well as an expert judgment for selecting information, data, and methods to better describe the processes. For the pesticide assessment, the definition of the application plan (i.e. timing and amount) in relation to the meteorological data was found to be extremely important, since it regulates processes, such as wash-off, infiltration, and surface runoff to be activated or not.

Model performances in simulating hydrology may be low in basins with temporary river systems^74,75^. In the dry season, the flow appears and disappears along the river network depending mostly on the lithology and geology of the area and secondarily on the rainfall regime, therefore, the assessment of the low flow and dry conditions is affected by a great uncertainty. In the Canale d’Aiedda, the SWAT model performed better for hydrology at gauge B (180 km^2^), with a larger drainage area, than for gauge A (37 km^2^). This is because some parameters have to be set at the basin scale. For instance, the parameter TRNSRCH.bsn, which controls the fraction of transmission losses from the main channel, can be calibrated only at the basin scale, therefore, it produced an overestimation at gauge A and an underestimation at gauge B^57^. In both gauges, the main peaks were well estimated, the normal flow was only slightly underestimated and the low flow was slightly overestimated. Difficulties in calibrating the low flow can be related to the constant values of discharge from WWTPs adopted as input that led to having an abiding simulated flow curve. Daily values of WWTP discharge may improve modelling results^45^. Moreover, uncertainties may be due to the parameterization of multiple factors (e.g. groundwater, topography, surface water exchanges with the subsoil, and management practices)^38^. To improve the simulation of the peak flow, the parameterization of the curve number (CN2.mgt) and the Manning’s roughness of the main channel (CH_N2.rte) had an important role. Indeed, these two parameters are strictly related to the runoff formation process and to the flow velocity^17^. Also, the parameterization of the fraction of percolation between the root zone and the deep aquifer (RCHRG_DP.gw) was very important in improving the simulation of the baseflow^17,61^.

The SWAT model simulates one pesticide at a time^62^, therefore, two different calibrations were performed, one for Cu and one for glyphosate, without changing hydrological parameters. Generally, sediments play a crucial role in the modeling of pesticide in surface water^76^. This was particularly true for Cu calibration, where the parameters related to sediment concentration and routing were fundamental. Indeed, the transport of Cu into the streamflow occurs predominantly in the suspended sediment absorbed form^77^. Also the soil adsorption coefficient parameter (SKOC.pest.dat) was sensitive. The value calibrated value for this parameter was slightly lower than the value reported by Serpa et al.^36^ Despite being soluble in water, glyphosate can easily also be adsorbed by soil and transported in suspended sediment^69,78^. Indeed, the soil adsorption coefficient parameter (SKOC.pest.dat), which controls the ratio between the pesticide concentration sorbed to the solid phase and the concentration in solution, resulted very sensitive in the calibration process. The calibrated value of this parameter is low respect the values reported in the IUPAC database, however in lines with the values reported the Environmental Protection Agency (EPA) database (https://comptox.epa.gov/dashboard/). The degradation half-life of the chemical on the soil (HLIFE_S.pest.dat) was also relevant for calibrating glyphosate. The calibrated value is slightly higher respect to the range reported in the IUPAC database. However, this parameter is highly variable and can range from a few days up to one or two years, depending on environmental conditions, such as temperature and soil humidity, and also on soil properties and agricultural practices^79–81^. The SWAT model performed satisfactory and good for Cu and glyphosate, respectively. However, it underestimated measured concentrations for both compounds. This could depend on the fact that SWAT does not simulate the drift loss which occurs during a pesticide application^44,82^, it is still unable to spatially reflect the fate of the drifted part of particles explicitly due to oversimplification^39^. Other sources of uncertainty, which could have influenced the results, are related to the quality and quantity of measurements (i.e. discrete data instead of continuous data) used for the calibration and to the temporal discrepancy between the calibration of the streamflow and the calibration of the pesticides concentration^57^. Similarly, the input data (i.e. application rates of the pesticides), which were derived from regulations or from the safety sheet, could be affected by a large uncertainty and they have had a key role in the modelling pesticides concentrations^39,83^. Therefore, the results could be improved by using data retrieved from farmers.

This study evidenced that Cu and glyphosate are used in extensive agriculture basins. The two main crops in the study area are vineyards and olive groves. Since the end of the nineteenth century, Cu has been used in vineyard productions as a fungicide^84^. According to investigations by Mackie et al.^85^, major applications of Cu as a fungicide are applied by spraying directly on the vine canopy to fight downy mildew (*Plasmopara viticola*) from May to August. On olive groves, it is a common practice to use a copper-based bactericide to provide protection against olive knots (*Pseudomonas savastanoi*), and further sprays, generally applied in the spring to improve control of other disease^86^. Frequent applications or a high application rate of Cu could be phytotoxic, especially when applying it at high temperatures or in dry weather^87–89^. Indeed, the Commission Implementing Regulation (EU) 2018/1981 limited the use of Cu at 28 kg ha^−1^ in a 7 years period^58^. In the Canale d’Aiedda, Cu showed high chronic ecotoxicological risk at the monthly scale in several river reaches. January and November were the months in which the higher number of reaches showed a TER < 10. This can be related both to the treatments and to the rainfall events. Indeed, the rainfall in November 2021 was 172.75 mm and in January 2021 was 46.55 mm. This study highlights that, in the study area, Cu delivered by the river (69.45 kg y^−1^) may accumulate in the sea environment affecting the aquatic ecosystem. Therefore, further studies are needed to investigate the accumulation of heavy metals in marine sediments of the Mar Piccolo.

Glyphosate is one of the most widely used non-selective herbicides in the world^90^. It acts only in post-emergence and is particularly effective when weeds are actively growing. The excessive dosage of glyphosate is of concern for the effects on the environment^9^. Although the leaching of glyphosate is limited, its continuous use can lead to a pollution of the shallow groundwater^91^. In surface waters, glyphosate converts very swiftly to its primary metabolite (i.e. AMPA) which is more persistent and harmful^92^, with a soil half-life higher than glyphosate. In 2016, the Italian Ministry of Health banned the use of glyphosate in agriculture and in the countryside, in all phases prior to wheat harvesting. Moreover, the WHO reclassified glyphosate as a probable carcinogen substance^93^. In 2019, the European Chemical Agency (ECHA) classified glyphosate as a dangerous and toxic substance to aquatic organisms^94^. This was also confirmed in 2022 by ECHA Committee for Risk Assessment (RAC) (https://echa.europa.eu/it/-/glyphosate-no-change-proposed-to-hazard-classification). Experiments demonstrated the genotoxicity on fish of Roundup^95,96^. In this context, monitoring activities and modeling the transport of glyphosate is needed to identify critical river reaches where mitigation measures could be implemented. Concerning glyphosate, the concentrations in river exceeded the EQS for surface waters. However, no reaches showed a chronic ecotoxicological risk related to the presence of glyphosate. In 2021, the analysis carried out at daily scale for glyphosate revealed that some reaches showed acute risk. In most of them the peak of concentration last 24 h, only a few cases showed a duration of 72 h. Also in the case of acute toxicity a relation between the peak of concentration and the rainfall was observed. The acute risk cases mainly occurred in April and November, when glyphosate treatments were generally applied. In the study area, glyphosate delivered by the contaminated river (71.94 kg y^−1^) may accumulate in the marine environment affecting the aquatic ecosystem, and the structure and function of aquatic communities. Therefore, further studies are needed to investigate the accumulation and impact of glyphosate in marine sediments of the Mar Piccolo.

Neither Cu nor glyphosate are identified as priority substances by the Directive 2013/39/EU^97^ nor are they included in the Watch List within the Commission Implementing Decision (EU) 2018/840^98^. The EU did not provide specific limits for these substances in surface waters^43^. However, also non-priority compounds can be potentially harmful to aquatic life^99,100^. Currently, the review process of EQSs is still ongoing. No specific EQS has been fixed for glyphosate, whereas for generic pesticides EQS is 0.1 μg l^−1^^70,71^.

The study highlighted that in the Canale d’Aiedda, some measures should be adopted to avoid inappropriate and intensive use of pesticides. Best Management Practices (BMPs) and Nature-based solutions (NBSs) should be promoted to reduce water and soil pollution. The methodological approach proposed in this work allows us to identify the areas under ecotoxicological risk within a river basin. However, the selection of the most suitable BMP or NBS requires further analysis which involves the local environmental policies and the economic feasibility for both the public and the private sector and to remove the barriers constituting a limit in the NBS adoption.

## Conclusions

Going beyond a case study, this work turns out to be fundamental to understanding the strengths and weaknesses of SWAT to simulate the fate and transport of pesticides in the surface runoff of an intermittent hydrological regime basin with a Mediterranean climate. Low rainfall, flow intermittence, and the limited data availability that characterize the Mediterranean basins make the modeling of pesticides a challenge. In this work, Cu and glyphosate were modelled with results ranging between satisfactory and good. The results, obtained after the calibration, show that the SWAT model was able to simulate Cu and glyphosate concentrations in a Mediterranean environment. However, improvements in the algorithm are desirable to correctly simulate wash-off threshold and drift in the Mediterranean environment. Moreover, improvement in the computation of the pesticide loads will be useful to better assess the model results. Indeed, SWAT provides only results at a reach scale and not at the HRU level.

This study highlighted that Cu and glyphosate are largely used in extensive agriculture basins. The methodological approach defined in this work, which was based on field measurements (streamflow and pesticide concentrations) coupled with modeling, proved to be able to able to evaluate the PEC and the ecotoxicological risk associated to the presence of Cu and glyphosate within the river network. In the Canale D’Aiedda case study, the results of monitoring and modeling activities revealed a chronic risk associated with the presence of Cu from November to April in several river reaches and acute risk associated to the presence of glyphosate in several reaches mainly in the wet season. The most important factor influencing the chronic risk for Cu were the combination of two factors: the high surface runoff and the Cu applications. The most important factor influencing the glyphosate peaks of concentration is the streamflow. The event based analysis shows a rapid dissipation of the substance.

Further studies are needed to investigate the ecotoxicological effect of multiple pollutants. This work lays the basis for future investigations (i.e. sustainable scenarios analysis) aiming at mitigate the concentrations of pesticides in surface water.

### Supplementary Information

Supplementary Information.

## Data Availability

All data generated or analyzed in the current study are available from the corresponding author on reasonable request.
