# Supplementary material for: Modeling pesticides and ecotoxicological risk assessment in an intermittent river using SWAT
Source: Sci Rep. 2024 Mar 16;14:6389. doi: 10.1038/s41598-024-56991-6 (PMC10944508; doi:10.1038/s41598-024-56991-6)
Supplement: Supplementary file 1 — Supplementary Information. [file 41598_2024_56991_MOESM1_ESM.pdf]

Modeling pesticides and ecotoxicological risk assessment in an intermittent river using SWAT

Marco Centanni[1], Giovanni Francesco Ricci[1*], Anna Maria De Girolamo[2], Francesco Gentile[1]

[1] Department of Soil, Plant and Food Sciences, University of Bari Aldo Moro, Bari, Italy
[2] Water Research Institute, National Research Council, Bari, Italy
[*]Corresponding Author: Giovanni Francesco Ricci

Graphical Abstract

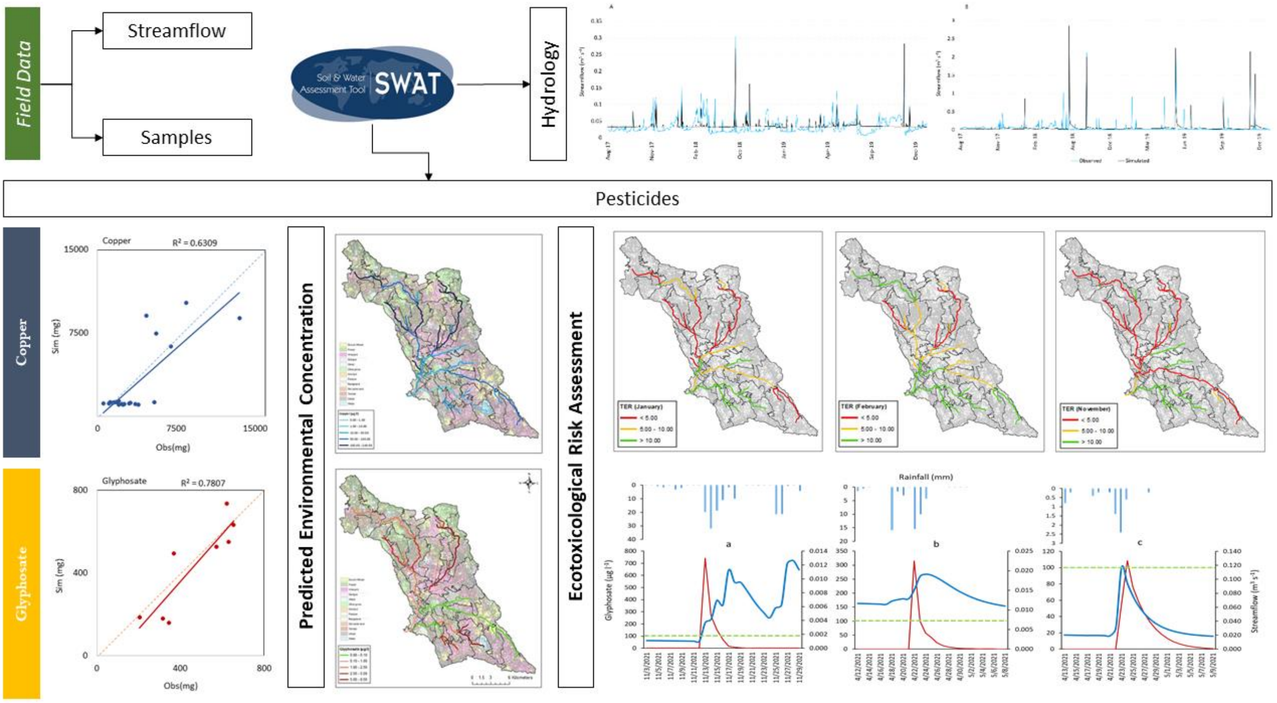

Table A1. Input data used for the SWAT model set-up, source and spatial resolution.

Input	Source	Resolution
Land use map	Puglia Region<br>(http://www.sit.puglia.it/portal/portale_cartografie_tecniche_tematiche/Cartografie%20Tematiche/UDS)<br>National Agricultural Census (http://censimentoagricoltura.istat.it/index.php?id=73)	1:5000<br>-
Digital Terrain Model (DTM)	Puglia Region<br>(http://www.sit.puglia.it/portal/portale_cartografie_tecniche_tematiche/Cartografie%20Tematiche/DTM)	
Soil map and database	Puglia Region (ACLA II Project, 2001, Personal communication)<br>JRC-ESDAC (https://esdac.jrc.ec.europa.eu/resource-type/datasets)	1:100000<br>500 × 500 m
Waste water tretament plants (WWTPs)	Apulian Water Authority (Personal communication)<br>Regional Agency for Environmental Protection<br>(http://www.arpa.puglia.it/web/guest/depuratori)	_
Meteorological data	Civil Protection Service - Puglia Region<br>(https://www.regione.puglia.it/web/protezionecivile/rete-di-monitoraggio-e-dati-meteo-idrometrici)<br>Regional Agency for Irrigation and Forestry Activities<br>(https://www.agrometeopuglia.it/osservazioni/mappa-stazioni-puglia)	
Agricultural practices	Interviews with farmers, agricultural advisors[47] and pesticide safety data sheet	_

Table A2. Acute risk assessment associated with the presence of copper: reach number (Fig. A1) and frequency of occurrence for fish.

Duration - 24h

Reach	1	2	3	4	5	6	7	8	9	10	11	12	13	14	15	16	17	18	19	20	21	22	23	24	25	26	27	28	29	30	31	32	33	34	35	36	37	38	39	40
January	2	2	1	4	2		2	3	6	3	5	3	1	3	3	2		2			3	4	1		1	1	1			1						4				
February				2	2			2	2		2	3		2	1	2					1	1			1			1												
March		4	4		1		3							1	1																									
April					2			1	1	1	1	1	1	1	1			1	1	1												1								
May																																								
June																																								
July																																								
August		1					1																																	
September		1	1				1	1											1																					
October																												1								1				
November	2	1	1				1		2	2	2	2	1	1	2	2			2									1		2		1		3		2				
December	2	3	2	2	1		2	2	1			2	1	1	1	1	1	1	1		2				2	2	1	1		1										1

Duration - 48h

Reach	1	2	3	4	5	6	7	8	9	10	11	12	13	14	15	16	17	18	19	20	21	22	23	24	25	26	27	28	29	30	31	32	33	34	35	36	37	38	39	40
January		1	2	1	2	1		1				2	2	2		2		2	2				1	1	1			2		1				1						1
February					1					1		1	1			1	1	1	1	1														1						
March	1							2						1																										
April				1					1		1						1																							
May																																								
June																																								
July																																								
August																																								
September														1																										
October																																								
November		2	1	1	1				1	1	1	1	1								1	1						1						1						
December		1	2	1	1			1								2	1	1		2					1	2		1								1				

Duration - 72h

Reach	1	2	3	4	5	6	7	8	9	10	11	12	13	14	15	16	17	18	19	20	21	22	23	24	25	26	27	28	29	30	31	32	33	34	35	36	37	38	39	40
January					1			1					1			1			1	1			1	1	1	2	2	1		1				1						
February						1											1						1	1		1	1			1										
March																																								
April						1						1	1	1		2	1		1																					
May																																								
June																																								
July																																								
August																																								
September																																								
October																																								
November	1		1			1		1				1	1			1	1	1																		2				
December																	1																							

Duration - 96h

Reach	1	2	3	4	5	6	7	8	9	10	11	12	13	14	15	16	17	18	19	20	21	22	23	24	25	26	27	28	29	30	31	32	33	34	35	36	37	38	39	40
January																	1																							1
February																																								
March																																								
April																																								
May																																								
June																																								
July																																								
August																																								
September																																								
October																																								
November					1	1							1	1			1											1												2
December							1							1					1																					1

Table A3. Acute risk assessment associated with the presence of glyphosate: reach number (Fig. A1) and frequency of occurrence for sediment dwelling organisms.

	Duration - 24h																																							
Reach	**1**	**2**	**3**	**4**	**5**	**6**	**7**	**8**	**9**	**10**	**11**	**12**	**13**	**14**	**15**	**16**	**17**	**18**	**19**	**20**	**21**	**22**	**23**	**24**	**25**	**26**	**27**	**28**	**29**	**30**	**31**	**32**	**33**	**34**	**35**	**36**	**37**	**38**	**39**	**40**
**January**		1			1											1																								
**February**																																								
**March**																																1	1					1	1	
**April**								1		1		1		1																										
**May**																																								
**June**																																								
**July**																																								
**August**		1																																						
**September**																																								
**October**																																								
**November**																																								
**December**																																								
	**Duration - 48h**																																							
Reach	**1**	**2**	**3**	**4**	**5**	**6**	**7**	**8**	**9**	**10**	**11**	**12**	**13**	**14**	**15**	**16**	**17**	**18**	**19**	**20**	**21**	**22**	**23**	**24**	**25**	**26**	**27**	**28**	**29**	**30**	**31**	**32**	**33**	**34**	**35**	**36**	**37**	**38**	**39**	**40**
**January**				1					1			1	1	1			1																							
**February**																																								
**March**																																								
**April**																																								
**May**																																								
**June**																																								
**July**																																								
**August**																																								
**September**																																								
**October**																																								
**November**																																								
**December**																																								
	**Duration - 72h**																																							
Reach	**1**	**2**	**3**	**4**	**5**	**6**	**7**	**8**	**9**	**10**	**11**	**12**	**13**	**14**	**15**	**16**	**17**	**18**	**19**	**20**	**21**	**22**	**23**	**24**	**25**	**26**	**27**	**28**	**29**	**30**	**31**	**32**	**33**	**34**	**35**	**36**	**37**	**38**	**39**	**40**
**January**																																								
**February**																																								
**March**																																								
**April**																1																								
**May**																																								
**June**																																								
**July**																																								
**August**																																								
**September**																																								
**October**																																								
**November**						1																																		
**December**																																								

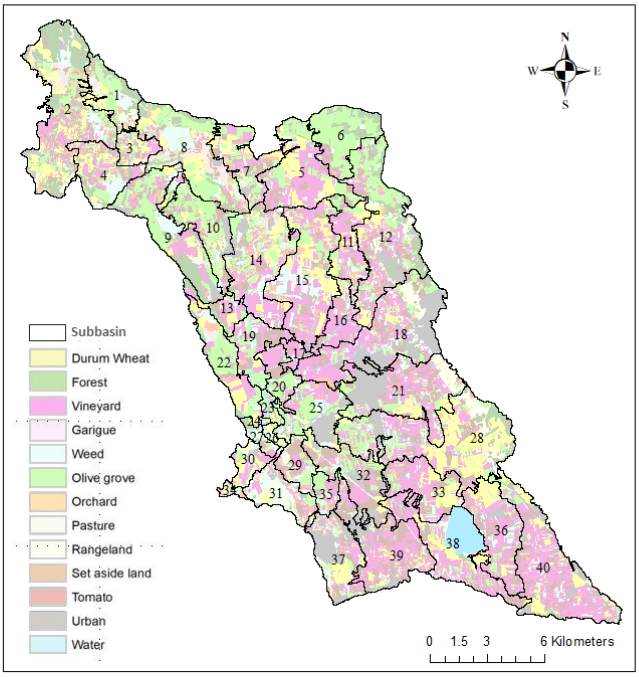

Figure A1. Subbasins and land uses for the Canale d'Aiedda (QGIS version 3.4.13. https://www.qgis.org/it/site/)

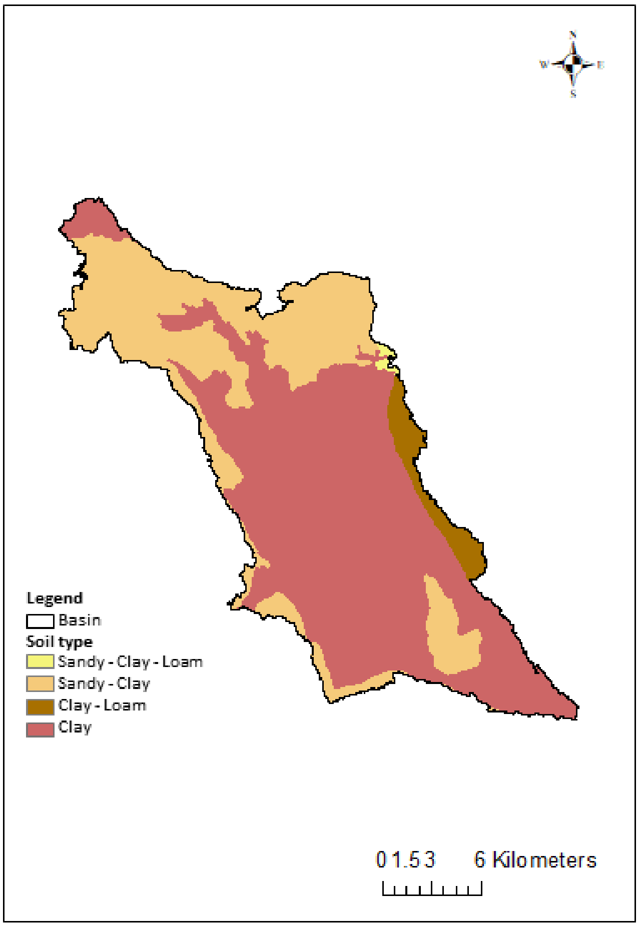

Figure A2. Soil map for the Canale d'Aiedda. (QGIS version 3.4.13. https://www.qgis.org/it/site/)

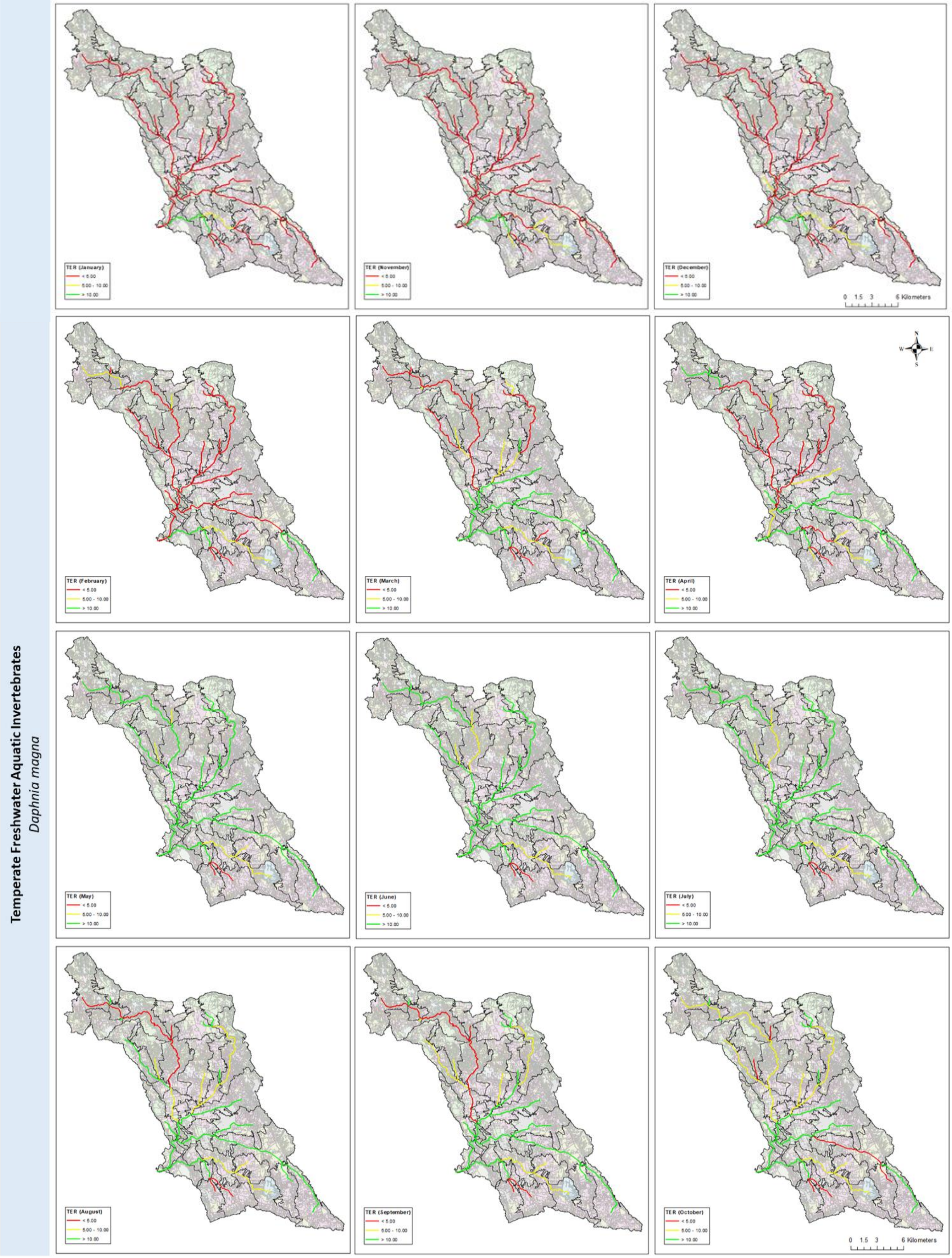

Figure A3. Maps of the monthly chronic Toxicity to Exposure Ratio (TER) for the copper for daphnia (Daphnia Magna) (2021). Red and yellow indicate the reaches under high exposure. (QGIS version 3.4.13. https://www.qgis.org/it/site/)

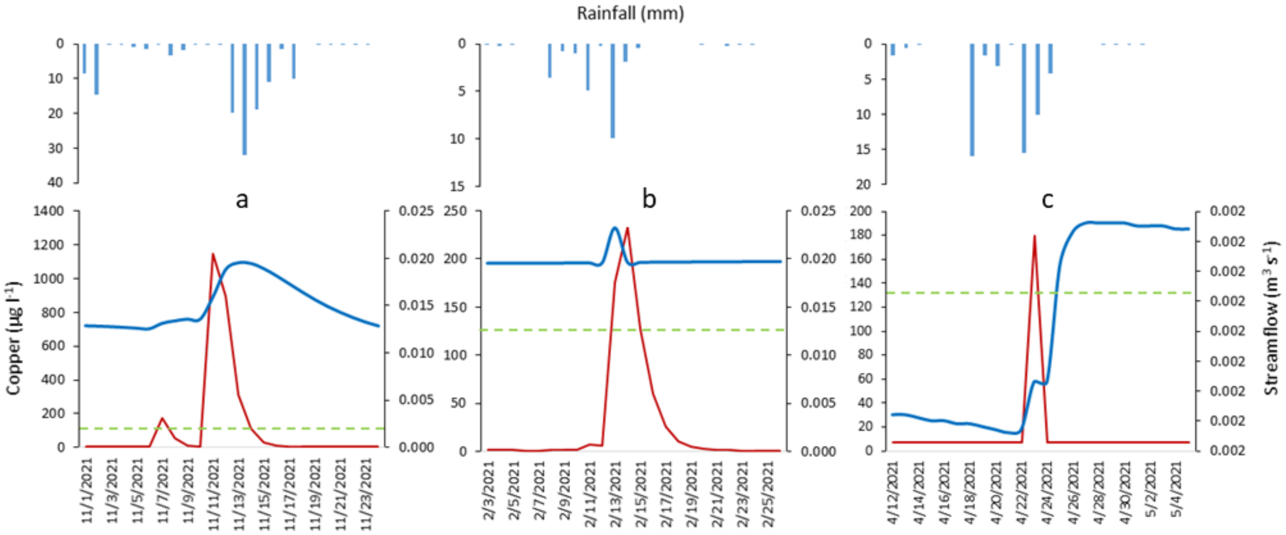

FigureA4. Concentration of glyphosate (red line), streamflow (blue line) and precipitation for three events occurred in 2021. The green line represents the concentration threshold for the acute risk for fish (Oncorhynchus mykiss). (a) reach n. 22 duration 72 h; (b) reach n. 34 duration 48 h and (c) reach n. 32 duration 24 h. The map of the reaches is reported in Fig. A1.
